# C_5_ Pentacle Structures: A Localization‐Delocalization Matrices Approach

**DOI:** 10.1002/open.202300277

**Published:** 2024-05-16

**Authors:** Julien Pilmé, Riccardo Spezia

**Affiliations:** ^1^ Sorbonne Université, L aboratoire de Chimie Théorique UMR 7616 CNRS 4 Place Jussieu 75005 Paris France

**Keywords:** Valence Bond Theory, Astrochemical molecules, DFT calculations, QTAIM, ELF

## Abstract

This article explores the possible presence of a pentacle valence bond structure in C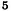 cyclic molecules. At this end, we have used quantum chemistry tools to elucidate the possible arrangement and the nature of chemical bonds within linear, cyclic, and three‐dimensional structures only formed by five carbon atoms. While the linear structure is clearly the most stable one, local minima were obtained for both bi‐ and three‐dimensional structures. Using the localization‐delocalization matrices approach, we characterize both the minimum linear structure and the cyclic ones. Interestingly, the linear structure is a combination of ionic and covalent bonds, albeit the four distances are almost identical, when using Density Functional Theory. For cyclic C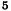 , the pentacle bonding arrangement emerges as a significant Lewis structure, indicative of an unusual formal configuration characterized by five intersecting C−C bonds. Our calculations show that this pentacle arrangement in cyclic C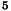 scheme is also present in the more known cyclo‐pentadienyl molecule.

## Graphical abstract

A pentacle valence structure is found in planar structure of C_5_ molecule and also in the more common cyclopentadienyl anion.

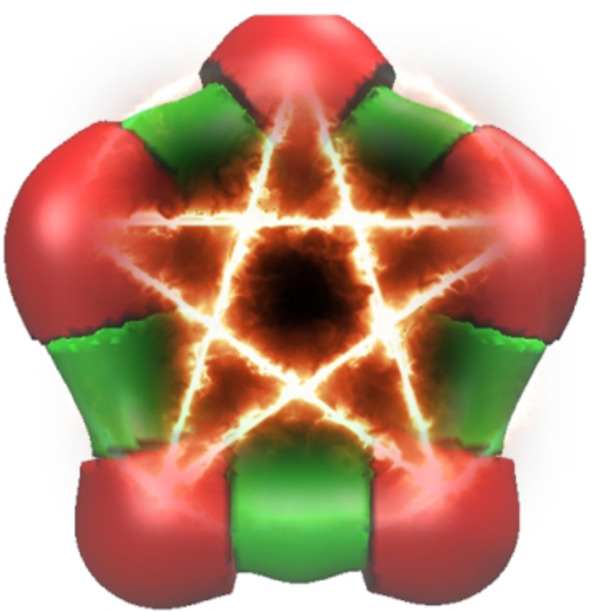

## Introduction

The ideas of regular geometries and symmetries had historically a crucial role in imaging and determining chemical structures. From a long time, the concept of valency, the nature of the chemical bond, is often related to structural considerations. One well‐known example is given by the Kekule's structure of benzene.^[1]^ The concept of resonance structures also comes from possible geometrical patterns in which chemical bonds can be disposed fullfilling the valence of atoms constituting the molecules.^[2]^ Another example of structure discovery motivated by geometrical considerations is the bicyclo[2.2.0]hexa‐2,5‐diene structure (an isomer of benzene) proposed theoretically by Dewar^[3]^ and synthesised by van Tamelen and Pappas almost one hundred year after.^[4]^ This alternative arrangement can be seen as coming from conceptual possible arrangement of bonds which are considered as “linkages between Daltonian atoms”.^[2]^

We can call this a geometry‐driven discovery which we can find in many other examples: from the simple water molecule,^[5]^ to transition metal complexes, in which group theory, leaded by symmetry, is the common way of describing them, to solid state chemistry (and physics) in which the symmetry is extended to periodicity and spatial groups are used.[^6^, ^7^]

In organic chemistry, triangles, squares, rectangles, pentagons, hexagons etc … made by carbon atoms are ubiquitous and they are used to simply rationalize bond making and breaking, thanks to the ability of carbon atoms to make multiple bonds. However, in organic chemistry in solution, carbon based structures are composed also by other atoms (at least H atoms), with the relevant exception of fullerenes. Interestingly, their structures were first suggested and observed experimentally only many years after.^[8]^

**Scheme 1:**
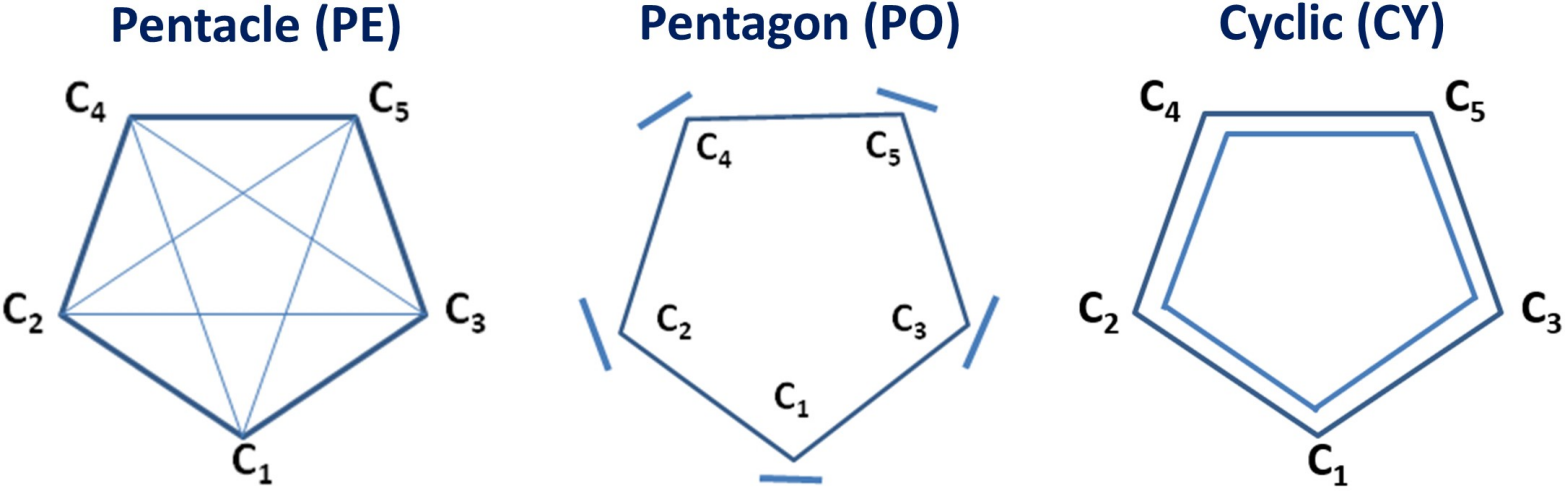
Three Lewis resonance structures of cyclic C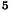 of D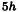 symmetry.

Amongst the possible geometrical figures, one seems missing to us: the pentacle. This is an old geometrical figure composed by five vertices all linked together. If, as usual, each segment is a chemical bond, this will generate a Lewis structure corresponding to a molecule composed by five carbon atoms (and no hydrogen or other atoms). This particular cyclic C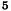 molecule can have, in principle, the bonds arranged in a way they form a pentacle motif (see Scheme 1). Here we have considered, in total, three reference Lewis structures for the cyclic form of C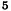 which have the same symmetry of a regular pentagon in the bonding pattern. First, the pentacle structure (PE) corresponds to a bonding scheme in which all five electron pairs are fully delocalized inside the cycle. Then, the pentagon structure (PO) corresponds to a bonding carbene‐like scheme where all five electron pairs are fully localized on the carbon atoms. Finally, the cyclic structure (CY) corresponds to a benzene‐like delocalization scheme where electrons are delocalized around the cycle.

We should remind that the C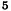 molecule has also an interest *per se*. In fact, it was studied theoretically and experimentally in particular due to its astrochemical importance,^[9]^ finding that the linear structure, belonging to the carbyne family,^[10]^ is the most stable one.[^11^, ^12^, ^13^, ^14^, ^15^, ^16^, ^17^, ^18^, ^19^, ^20^, ^21^] Five atoms can be arranged in different ways, linear, cyclic or with a three‐dimensional arrangement.^[22]^ In the case of C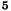 , other than the aforementioned linear structure, bi‐ and three‐dimensional geometries were identified, as stable singlet or triplet states.[^12^, ^17^] However, no detailed analysis of the chemical bond nature within these structures was conducted in previous studies. In the present contribution, we studied in detail the properties of chemical bonds of $C_{5}$ structures, in particular the most stable linear carbyne and the two‐dimensional structures which can in principle have a pentacle Lewis structure.

We considered the different possibilities for C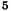 to be arranged in space: as chain, as a ring and as a branched structure in order to provide also a general picture on the possible arrangement of C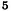 , even for non‐cyclic structures. Furthermore, the LDM approach when applied to the linear structure can be compared with the more standard QTAIM based approach.[^23^, ^24^] To this purpose, we used theoretical chemistry and in particular Density Functional Theory (DFT) within the well‐parameterized M06‐2X functional^[25]^ to determine the different geometries and to scrutiny the possibility that such particular resonance structure is present in C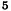 molecule. Results obtained with DFT could be compared with previous data mainly based on wave‐function approaches, thereby offering a degree of reliability in using DFT for larger carbon chains, where highly correlated methods cannot be afforted. Furthermore, we have also quantified the contribution of pentacle resonance structure in the well‐known cyclopentadienyl, C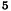 H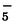 , structure.

## Theory

### Quantum Chemical Topology

In this paper, we assume that the reader is familiar with the quantum chemical topology (QCT) of scalar fields because numerous presentations of the methodology have already been published in the literature.[^24^, ^26^] Briefly, QCT is devoted to answer general questions about the chemical bonding in molecules and solids, and predict or explain chemical reactivity trends. This approach relies on the theory of gradient dynamical systems that enables a partitioning of the molecular space into basins. The most used one is the electron density, giving rise to the quantum theory of atoms‐in‐molecules (QTAIM).^[27]^ These basins being only atomic ones, a topological atom can be defined as the union of a nucleus with its associated electron density basin. Another widely used function is the Electron Localization Function (ELF) usually interpreted as a signature of the distribution of electron‐pairs in the molecular space.^[28]^ The ELF topology depicts some non‐atomic valence basins in addition to valence and core basins surrounding nuclei with atomic number Z> 2. In all the cases, the basins are delimited by zero‐flux surfaces and the integration of the electron density over each basin directly provides its corresponding population. For example, the QTAIM atomic charge, q , is calculated by subtracting the electron population of the topological atom. Introduced by Bader and Stephens^[29]^ and later recovered by Fradera et al.^[30]^ the delocalization index (δ ) is a measure of the electron‐sharing between two atoms and can be compared to other bond order indices.

### Molecular Similarity

Molecular similarity is a fundamental concept in chemistry. It is crucial to many aspects of chemical reasoning and for example, it stands as a cornerstone assumption in medicinal chemistry.^[31]^ Conversely, dissimilarity plays a significant role in an expanding array of applications particularly in combinatorial chemistry or for the the virtual screening.^[32]^ Several types of mathematical methods can be used to represent the molecular dissimilarity. Among these various approaches, the electron localization‐delocalization real and symmetric matrix (LDM), is a representative graph of a molecule where all atoms (vertices) are interconnected by the QTAIM delocalization indices.^[33]^ The summation of off‐diagonal elements of a LDM corresponds to the total delocalized population of the molecule. The trace of a LDM (N×N ) is the total localized electron population of the molecule:

(1) $$LDM=\left(\begin{array}{ccccc} & \lambda_{A} & \delta_{AB}/2 & ... & \delta_{AN}/2\\ & \delta_{BA}/2 & \lambda_{B} & ... & \delta_{BN}/2\\ & .... & .... & ... & ....\\ & \delta_{NA}/2 & \delta_{NB}/2 & ... & \lambda_{N} \end{array}\right)$$

where $\lambda_{i}$ are the localization indices and $\delta_{ij}$ the delocalization indices (positive when atoms interact). Although several limitations have been identified, the LDM matrix is a powerful tool to measure the similarity/dissimilarity of different molecules themselves or as a predictor in QSAR methods.^[34]^ There does not exist a unique way to compare matrices. However, the Euclidian‐type norm (termed as Frobenius distance) is commonly used to evaluate the dissimilarities between matrices. The dissimilarities between two molecules A and B can be then evaluated using the Frobenius distance d(A,B) applied to the LDM:

(2) $$d\left(A,B\right)=\sqrt{\sum_{i,j}{\left|a_{ij}-b_{ij}\right|}^{2}}$$

Where $a_{ij}$ and $b_{ij}$ are the corresponding matrix elements of LDMs of A and B molecules respectively. Smaller the value of the distance, more similar are the two molecules. With this point, the Frobenius distance can be used for measuring the similarity between structures.[^35^, ^36^] For convenience, it is also possible to define weights associated to LDM structures. The weights, restricted to [0, 1], can be easily computed from any number N of Frobenius distances d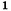 , d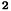 ,..d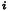 ,..d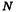 as follows:

(3) $$\omega_{i}=\;\frac{1}{1+\sum_{j\neq i}^{N}\frac{D_{i}}{D_{j}}}$$

where $D_{i}$ =d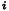 +ϵ , ϵ being an arbitrary small threshold which prevents the division by zero and has been set to 10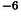 in this study. When $D_{i}$ goes to zero, $\omega_{i}$ goes to one. Conversely, if $D_{i}$ become large, $\omega_{i}$ goes to zero. In this work, the Frobenius distances $d_{i}$ have been calculated for optimized DFT structures with respect to formal Lewis resonant structures $d_{j}$ of the same symmetry used as references here.

## Results and Discussion

### Structural Parameters

We consider three arrangements of C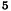 : linear 1D (or more in general a segment), 2D (a cyclic structure) and 3D (for which we considered different spatial arrangements). The most stable geometry corresponds to a linear 1D structure exhibiting a D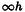 symmetry (X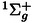 ) in agreement with some previous studies.[^12^, ^13^, ^16^, ^17^, ^21^, ^37^] The structure was also detected in the circumstellar shell of IRC+10216.[^38^, ^39^] We have then considered other arrangements for a closed shell singlet configuration.

**Figure 1:**
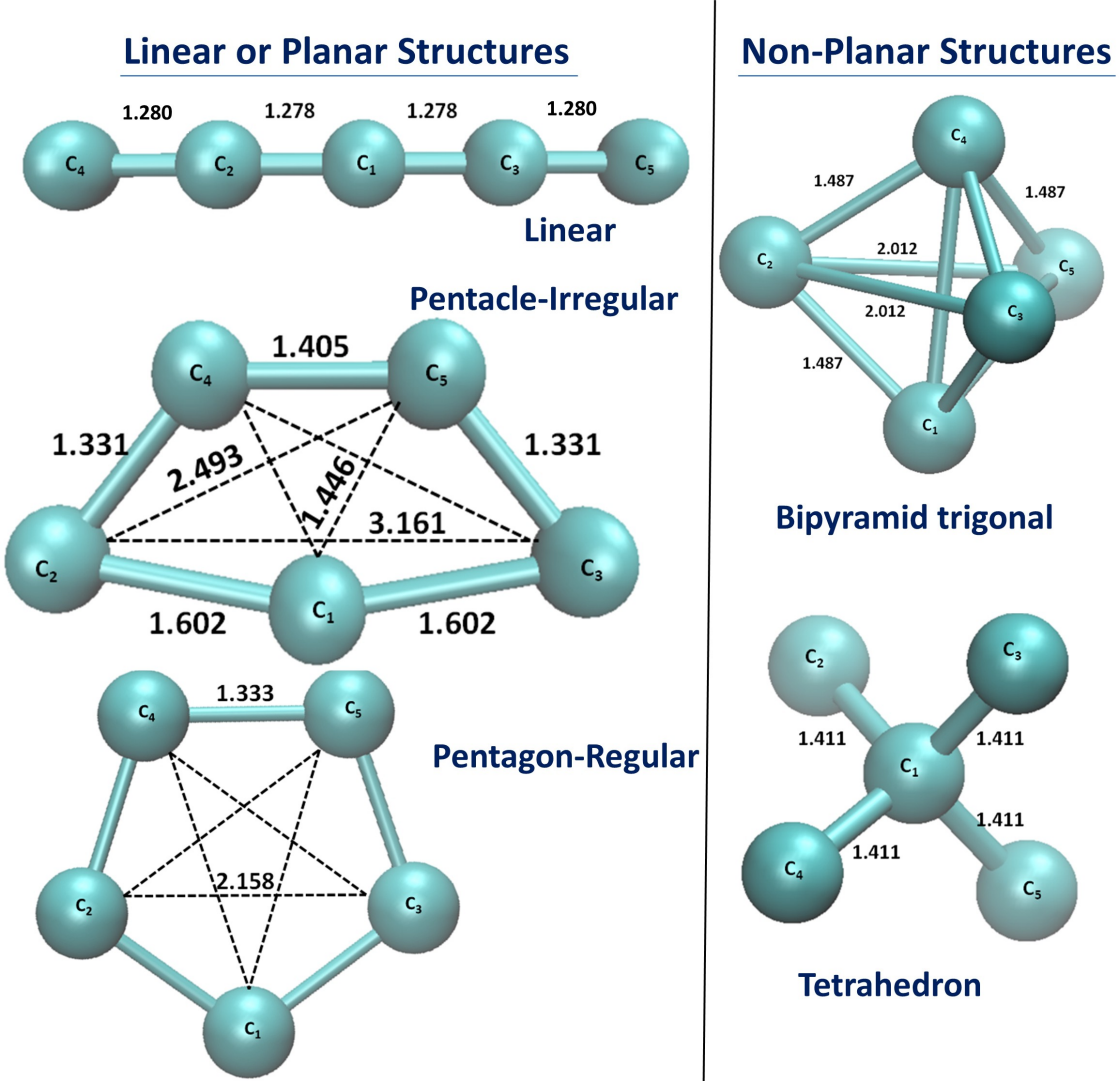
Optimized C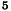 Structures. Geometries have been optimized at the M06‐2X/aug‐cc‐pVTZ level of theory. Distances are given in Å.

Even limiting ourselves to singlet states, we found one minimum for 2D cyclic (a planar C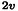 ) and one for 3D structures (a bipyramid trigonal, D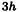 ). All the geometries are shown in Figure 1 while structural and energetic results are gathered in Table 1. More details on geometries, vibrational frequencies and rotational constants for all the minima are detailed in SI, Tables S2–S5. The 2D cyclic minimum (termed as Pentacle‐Irregular) corresponds to a distorted pentagon where the bonds are not all the same and with a C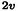 symmetry and it is 43.8 kcal/mol higher in energy than the linear structure. We also searched for a regular pentagonal structure (termed as Pentagon‐Regular) with a D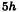 symmetry where all bonds and angles are equal. However, we can find only one geometry and it turned out to be a saddle point. Notably, we obtained two imaginary frequencies, one corresponding to an out‐of‐plane motion consistent with a Jahn‐Teller distortion, while the other one leads to the Pentacle‐Regular structure.

**Table 1:** Structural and energy parameters of C structures optimized at the M06‐2X/aug‐cc‐pVTZ level of theory. The carbons numbering is given in Figure 2.

Molecule C 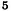	Symmetry	Δ E	R 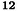	R 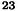	R 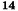	B
		(kcal/mol)	(Å)	(Å)	(Å)	(GHz)
Linear	D 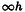	0.0^[a]^	1.278	2.556	2.558	2.574066
			1.3057^[d]^	2.6114^[d]^	2.6221^[d]^	
			1.2819^[e]^	2.5638^[e]^	2.57149^[e]^	2.5506^[e]^
						2.5704^[f]^
Pentacle‐Irregular	C 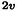	43.8^[a]^	1.602	3.161	1.446	
Bipyramid trigonal	D 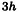	80.6^[a]^	1.487	2.012	1.857	
Pentagon‐Regular	D 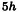	174.3^[b]^	1.333	2.158	2.158	
Tetrahedron	T 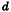	259.5^[c]^	1.411	2.305	1.411	
C 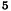 H 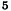 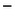	D 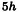		1.408	2.279	2.279	

[a] Minimum structure. [b] Second‐order saddle point. [c] Saddle point with three degenerated imaginary frequencies. [d] CCSD(T)/cc‐pVDZ calculations from Ref. [15]. [e] CCSD(T)/cGTOs from Ref. [11]. [f] B3LYP/aug‐cc‐pVTZ from Ref. [17].

We should notice that in previous studies, a cyclic minimum structure was obtained as triplet state using B3LYP/6‐31G(d) optimization followed by single point CCSD(T)/aug‐cc‐pVDZ energy calculation^[16]^ as well as in previous MP2/D95* calculations.^[13]^ Previous calculations using MP4/6‐31G(d) method, have found two single state minima: a cyclic isomer and a bipyrimid trigonal structure, being higher than the linear one by 60 and 95 kcal/mol, respectively.^[12]^ Similar behaviour are obtained by Roos and co‐workers from B3LYP/6‐31G(d) optimizations, even if the C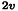 structures is now 76 kcal/mol higher than the linear carbyne.^[17]^ We should notice that the C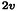 structures obtained in previous studies are not exactly what we called Pentacle‐Irregular, but, since they bear the same symmetry, they can be obtained simply by moving two C‐atoms in the plane. For example, Pentacle‐Irregular can become the $t-C_{5}$ structure of Masso et al.^[17]^ by moving C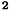 and C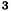 atoms apart, such that C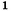 , C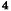 and C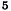 will form a triangle.

Moving to 3D arrangement, we obtained a stable structure which corresponds to a bipyramid trigonal one (D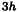 symmetry), being 80.6 kcal/mol higher in energy than the linear structure, while the tetrahedral structure (T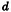 symmetry) is a saddle point. Previously, calculations using wave‐function based methods (MP2 and MP4) found a similar structure but 91–95 kcal/mol higher in energy than the carbyne,[^12^, ^13^] and 101 kcal/mol using B3LYP.^[17]^ From a connection with simple concepts that are familiar to chemists, the valence‐shell electron pair repulsion model (VSEPR) model^[40]^ proven its indisputable utility for helping chemists to rationalize molecular architectures somehow with hands. Interestingly, the C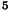 arrangement deviates from the standard VSEPR prediction. Indeed, while the predicted VSEPR‐type suggests a tetrahedral three‐dimensional arrangement (AX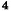 ≡ CC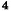 ), it is worth noting that this latter tetrahedral structure is not a minimum and it is more than 250 kcal/mol above the linear structure.

### The Linear Structure

The D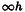 structure is the global minimum of C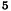 and it was previously theoretically studied.[^11^, ^12^, ^13^, ^15^, ^16^, ^17^] Our results are the first using novel DFT functionals with extended basis set and are thus compared with previous studies which were mostly wave‐function based (in some cases the geometries were obtained using DFT with relatively small basis set). Some comparison are shown in Table 1 where also the rotational constant is reported, while a more exhaustive comparison is reported in Table S2 of the SI. Notably, M06‐2X (as well as PBE0) found the internal C−C bond slightly shorter than the external one, as in CCSD(T) calculations. Furthermore, we report (see Table S3) the vibrational frequencies obtained with the two functionals, which agree well with previous calculations[^15^, ^17^] and experiments.[^18^, ^19^, ^20^]

**Figure 2:**
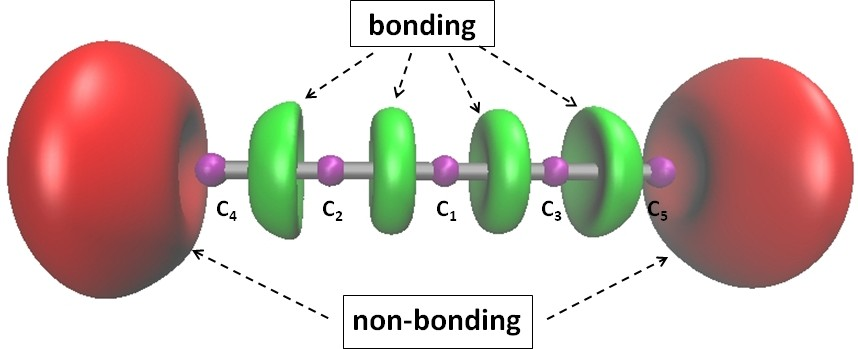
ELF localization domains (isosurface=0.8) for the C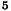 linear structure computed at the M06‐2X/aug‐cc‐pVTZ level of theory. Color code: magenta for core basins, red for valence non‐bonding basins and green for bonding basins.

In this work, our main aim is, however, to characterize the Lewis structures relevant for C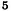 species and in particular to assess the validity of expected Lewis structure of the ground state of the C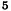 molecule regarding the geometry and its electron pair domains. With ten electron pairs, the expected Lewis structure gathers eight bonding domains (four σ bonds and four π bonds) together with two lone pairs belonging to the σ system and located around the terminal carbon atoms (carbene like structure). As shown in Figure 2, the occurrence of ELF domains in the linear geometry perfectly matches with the Lewis prediction on the location of domains.

In addition to core basins, the ELF topology yields several valence basins. Among them, two non‐bonding basins accounting for the carbon lone‐pairs are located on the right and on the left of the linear structure. Four bonding basins, accounting for the covalent electron‐pairing are identified in this structure. However, as shown in Table 2, the computed QTAIM charges and delocalization indices do not match with a symmetric structure where all the C−C bonds are almost identical.

**Table 2:** QTAIM analysis. Atomic populations ($\bar{N}$ ) and delocalization index (δ ) in electrons. The carbons numbering is given in Figure 1

Linear C 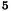	$\bar{N}$ (e)	δ (e)
C 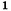	6.53	C 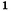 −C 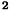 : 1.20
C 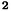	6.17	C 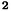 −C 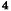 : 1.94
C 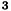	6.17	C 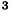 −C 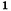 : 1.20
C 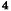	5.57	C 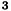 −C 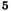 : 1.94
C 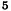	5.57	

**Scheme 2:**
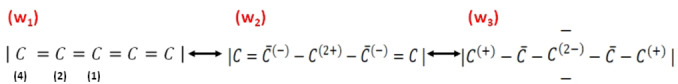
Proposed Lewis resonance scheme for the linear C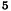 global minimum.

Indeed, the delocalization index is close to 2 for the C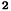 −C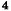 and C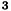 −C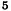 bonds while it is close to 1 for the C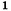 −C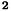 and C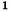 −C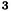 bonds. These results align with negative QTAIM charges for the central atoms (C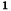 , C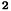 , C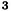 ) and positive for the terminal atoms (C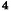 and C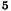 ). Comparing the ELF and QTAIM data leads us to propose a resonance Lewis scheme swinging between covalent and ionic structures where localized lone pairs (4 σ electrons) are considered at the terminal carbons. In addition, all 8 π electrons can be delocalized in each structure. The resulting resonance scheme is given in Scheme 2 and takes into account all of these results.

To give a flavor of the potential weights of these selected Lewis structures, we can use the QTAIM populations, as done by Silvi and others.^[41]^ For symmetry reasons, we can only get three atomic charges and thus, we can access to three different weights as shown in Scheme 2. Therefore, we consider the three structures with their associated weights (given in parenthesis) in agreement with the QTAIM populations: a pure covalent ($\omega_{1}$ ) and two ionic symmetric structures ($\omega_{2}$ and $\omega_{3}$ ). Let's consider the case of the C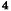 atom. C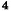 has a formal population of 6 electrons in the structures $\omega_{1}$ and $\omega_{2}$ , whereas it has a formal population of 5 electrons in $\omega_{3}$ . Since the QTAIM population $\bar{N}\left(C_{4}\right)$ is 5.5661e, the following equation must be satisfied: 6 $\omega_{1}$ +6 $\omega_{2}$ +5 $\omega_{3}$ =5.5661e. We can follow the same way for the carbons C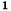 and C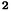 which leads to the following system of equations:

(4) $$\left\{\begin{array}{ccc} C_{4}:6\;\omega_{1}+6\;\omega_{2}+5\;\omega_{3}\; & =\; & \bar{N}\left(C_{4}\right)=5.566\;\\ C_{2}:6\;\omega_{1}+7\;\omega_{2}+6\;\omega_{3}\; & =\; & \bar{N}\left(C_{2}\right)=6.167\;\\ C_{1}:6\;\omega_{1}+4\;\omega_{2}+8\;\omega_{3}\; & =\; & \bar{N}\left(C_{1}\right)=6.531\; \end{array}\right.$$

Where $\bar{N}\left(C_{i}\right)$ is the QTAIM population (electron) of the carbon C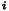 . The resulting weights are $\omega_{1}$ =0.40, $\omega_{2}$ =0.17 and $\omega_{3}$ =0.43. The strong weight of the covalent structure is in line with the trend observed for other typical linear carbon systems.^[42]^ However, we can note that the full‐ionic weight $(\omega_{2}$ +$\omega_{3}$ ) of 0.6 is actually predominant. It is possible to compute the weights of w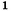 , w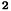 and w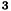 structures using the corresponding LDM matrices of D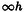 symmetry which can be defined as follows:

(5) $$w_{1}=\left(\begin{array}{ccccc} 4 & 1 & 1 & 0 & 0\\ 1 & 4 & 0 & 1 & 0\\ 1 & 0 & 4 & 0 & 1\\ 0 & 1 & 0 & 4 & 0\\ 0 & 0 & 1 & 0 & 4 \end{array}\right)$$

(6) $$w_{2}=\left(\begin{array}{ccccc} 3 & 0.5 & 0.5 & 0 & 0\\ 0.5 & 5.5 & 0 & 1 & 0\\ 0.5 & 0 & 5.5 & 0 & 1\\ 0 & 0.5 & 0 & 5.5 & 0\\ 0 & 0 & 1 & 0 & 5 \end{array}\right)$$

(7) $$w_{3}=\left(\begin{array}{ccccc} 7 & 0.5 & 0.5 & 0 & 0\\ 0.5 & 5 & 0 & 0.5 & 0\\ 0.5 & 0 & 5 & 0 & 0.5\\ 0 & 0.5 & 0 & 4.5 & 0\\ 0 & 0 & 0.5 & 0 & 4.5 \end{array}\right)$$

The Frobenius distances of the linear structure (see SI) with respect to w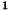 , w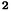 and w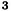 can be calculated together with the corresponding weights given by Eq. 3. The resulting weights are $\omega_{1}$ =0.42, $\omega_{2}$ =0.26 and $\omega_{3}$ =0.32. Again, we can note that the'full‐ionic’ weight ($\omega_{2}$ +$\omega_{3}$ =0.58) is actually predominant and its weight is similar to the 0.6 found by QTAIM‐based analysis. This is also in line with a carbyne‐like structure where the terminal carbons are rather electron deficient while the charges of central carbons are rather negatives. We note that the values of delocalization indices (see Table 2) remain surprising in view of the linear structure where all C−C distances are almost identical. As indicated by the computed weights, this is probably due to a subtle resonance picture that highlights a balance between totally symmetrical covalent and ionic symmetric structures.

### The Cyclic Structures

As discussed previously, we consider two cyclic C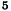 structures, the Pentacle‐Irregular, which is a local minimum, and the Pentagon‐Regular, which is a saddle point, but the lowest energy structure with the D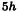 symmetry. Details on vibrational frequencies and rotational constants for the minimum energy structure (Pentacle‐Irregular) are reported in SI. This structure was never reported and these values could be useful for further characterization.

**Figure 3:**
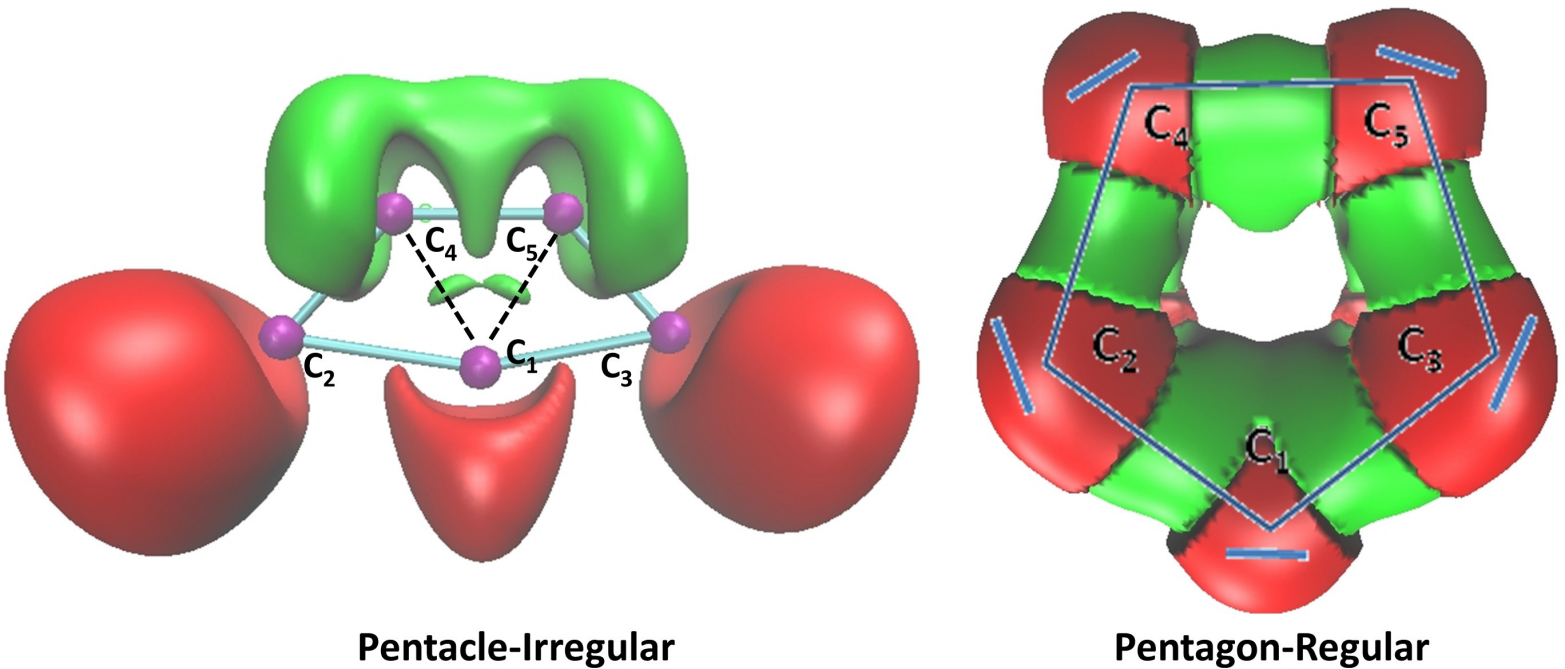
ELF localization domains (isosurface=0.8) for the C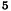 cyclic structures calculated at the M06‐2X/aug‐cc‐pVTZ level of theory. Color code: magenta for core basins, red for valence non‐bonding basins and green for bonding basins.

We now focus on the two cyclic geometries, which can have potentially a pentacle Lewis structure. As shown in Figure 3, the ELF topology of both Pentacle‐Irregular (minima) and Pentagon‐Regular (saddle point) yields several valence basins.

Among them, non‐bonding basins accounting for the carbon lone‐pairs are located on only three carbons for Pentacle‐Irregular while all carbons are involved for Pentagon‐Regular. For the latter, this aligns perfectly with the formal Lewis structure Pentagon (see Figure 3) where no inner C−C bonds are found. Interestingly, two similar bonding basins accounting for the covalent electron‐pairing are identified inside this Pentacle‐Irregular which exhibits rather striking resemblances to the pentacle formal structure (see Figure 3).

In order to quantitatively evaluate the similarity between the DFT‐optimized structures and the formal Lewis structures (pentacle and pentagon), we propose to use the LDM formalism previously detailed in the theory section. Note that the way used in the previous section to build a resonant system of equations from QTAIM populations cannot be applied to the cyclic forms because all the formal atomic populations in both structures (pentacle and pentagon) are equal to 6 electrons (see Scheme 1). However, we can use the LDM matrices of the formal C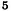 Pentacle (PE), Pentagon (PO) and Cyclic (CY) structures of D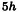 symmetry, which are defined as follows:

(8) $$\mathrm{PE}=\left(\begin{array}{ccccc} 4 & 1/2 & 1/2 & 1/2 & 1/2\\ 1/2 & 4 & 1/2 & 1/2 & 1/2\\ 1/2 & 1/2 & 4 & 1/2 & 1/2\\ 1/2 & 1/2 & 1/2 & 4 & 1/2\\ 1/2 & 1/2 & 1/2 & 1/2 & 4 \end{array}\right)$$

(9) $$\mathrm{PO}=\left(\begin{array}{ccccc} 5 & 1/2 & 1/2 & 0 & 0\\ 1/2 & 5 & 0 & 1/2 & 0\\ 1/2 & 0 & 5 & 0 & 1/2\\ 0 & 1/2 & 0 & 5 & 1/2\\ 0 & 0 & 1/2 & 1/2 & 5 \end{array}\right)$$

(10) $$\mathrm{CY}=\left(\begin{array}{ccccc} 4 & 1 & 1 & 0 & 0\\ 1 & 4 & 0 & 1 & 0\\ 1 & 0 & 4 & 0 & 1\\ 0 & 1 & 0 & 4 & 1\\ 0 & 0 & 1 & 1 & 4 \end{array}\right)$$

We have computed the delocalization indices and corresponding LDM matrices for the different DFT cyclic structures and measure the Frobenius distances with respect to three reference formal matrices of same D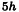 symmetry, as explained in the Theory section. For example, the following LDM matrix corresponds to the Pentagon‐Regular molecule is:

(11) $$\left(\begin{array}{ccccc} 4.008 & 0.853 & 0.853 & 0.142 & 0.142\\ 0.853 & 4.008 & 0.142 & 0.853 & 0.142\\ 0.853 & 0.142 & 4.008 & 0.142 & 0.853\\ 0.142 & 0.853 & 0.142 & 4.008 & 0.853\\ 0.142 & 0.142 & 0.853 & 0.853 & 4.008 \end{array}\right)$$

**Figure 4:**
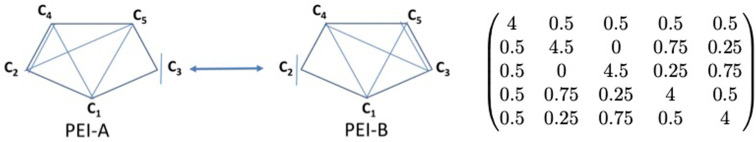
Average Pentacle‐Irregular (PEI) LDM computed as APEI=$\frac{1}{2}$ (PEI−A+PEI−B).

As the DFT Pentacle‐Irregular displays two short C−C and two long C−C distances, we also need to consider an additional average Pentacle‐Irregular structure (APEI), which is provided in Figure 4. This latter LDM involves a partial inner delocalized density in agreement with the ELF localization domains provided in Figure 3, where two small inner disynaptic ELF basins are observed along the C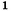 −C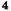 and C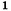 −C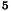 bonds.

All the results are reported in Table 3 where we also report the distance for PE, PO and CY as reference as well as what obtained from C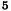 H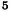 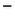 .

**Table 3:** Frobenius distances and corresponding weights of PE, PO, CY and APEI Lewis structures for different cyclic C molecules.

Molecule C 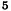	d 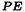	d 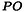	d 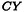	d 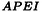 ^[a]^	$\omega_{PE}$	$\omega_{PO}$	$\omega_{CY}$	$\omega_{APEI}$ ^[a]^
D_2h_ Structures								
Pentacle (PE)	0.0	2.738	2.236		1.0	0.0	0.0	
Pentagon (PO)	2.738	0.0	2.738		0.0	1.0	0.0	
Cyclic (CY)	2.236	2.738	0.0		0.0	0.0	1.0	
Pentagon‐Regular	1.588	2.524	0.650		0.25	0.15	0.60	
C 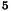 H 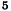 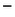	1.677	2.018	2.524		0.40	0.34	0.26	
C_2*v*_ Structures								
Average Pentacle Irregular (APEI)	1.224	2.236	1.870	0.0	0.0	0.0	0.0	1.0
Pentacle‐Irregular	1.441	2.017	2.049	0.868	0.25	0.17	0.17	0.41

[a] Average Pentacle Irregular (APEI) as shown in Figure 4.

Results are reported in Table 3 and divided following the symmetry of the different structures: D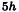 for Pentagon‐Regular and C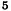 H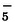 and C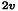 for Pentagle‐Irregular. Surprisingly, the pentacle Lewis structures make an important contribution in both DFT structures, Pentagon‐Regular and Pentacle‐Irregular. Notably, structures with *crossing bonds*, i. e. PE and APEI, show an important contribution, with a weight exceeding 60 % ($\omega_{PE}$ + $\omega_{APEI}$ ) for Pentacle‐Irregular and also exceeding 40 % ($\omega_{PE}$ ) for the well‐known C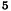 H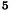 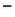 molecule. Note that for Pentagon‐Regular, it was rather difficult to characterize the contribution of the pentacle Lewis structure from only ELF localization domains (see Figure 3) since none inner C−C bonding basin has been found inside the pentagon. These results illustrate that the rationalization of the electronic structure of cyclic structures needed a thorough analysis of a set of delocalization indices revealing the role of inner delocalized density in the pentacle cycle. Finally, based on the weights obtained from the LDM matrix analysis, the aromatic character of Pentacle Irregular structures appears to be quite comparable to that of the cyclopentadienyl anion, which exhibits maximum aromaticity with five equivalent bonds and a totally delocalized five‐membered ring.^[43]^

## Conclusions and Outlooks

It is remarkable how small and simple systems, such as the C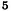 molecule, still offer a rich playground for a better understanding of usual concepts, such as the resonance. In particular, when the five carbon atoms are arranged in a ring, we obtained that a formal structure with bonds passing inside the ring is an important one: this intriguingly corresponds to a pentacle, a particular geometrical motif which was not considered before. This particular bonding pattern is intriguing because the bonds formally *cross* each other. Notably, this pattern is not negligible also in the hydrogenated form, the less exotic C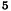 H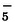 molecule. While a pentacle is possible only in C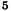 systems, it will be interesting to know if other *crossing bonds* structures are possible resonance structures and how this is related (or not) to their reactivity.

Furthermore, we have found that modern DFT functionals with extended basis sets are able to obtain the linear carbyne structure as the most stable minimum, and to locate stable a 2D cyclic structure never considered and/or obtained before, and the well documented 3D bipyramid trigonal structure. Thus, it would be possible to increase the carbon length using DFT and then see how (and if) 2D and 3D geometries become more and more stable and if such crossing bonds are responsible to their stabilization of other structures with growing number of carbon atoms. This question will surely deserve more investigations.

## Computational Details

The M06‐2X hybrid functional^[25]^ level with the Gaussian 16 software was used for all calculations.^[44]^ The standard all‐electron aug‐cc‐pVTZ basis set was used for all atoms.^[45]^ The C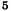 linear ground state structure was fully optimized without symmetry constraints. In a first step, local minima geometries were approached through symmetry constrained calculations. For instance, D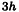 was used for trigonal bipyramid. We then fully optimized the geometries using the local minimum‐energy structures as initial guesses. Each minimum displays only positive eigenvalues. Optimizing other structures with different symmetry constraints always resulted in higher order saddle points, with two or three imaginary frequencies for second‐order and third‐order saddle points, respectively. Only the single states for each geometry were considered. The quantum chemical topology analyses and LDM matrices have been performed using the TopChem2 program package.[^46^, ^47^]

## Conflict of Interests

No conflict of interest to declare.

1

## Supporting information

As a service to our authors and readers, this journal provides supporting information supplied by the authors. Such materials are peer reviewed and may be re‐organized for online delivery, but are not copy‐edited or typeset. Technical support issues arising from supporting information (other than missing files) should be addressed to the authors.

Supporting Information

## Data Availability

The data that support the findings of this study are available from the corresponding author upon reasonable request.
