# Supplementary material for: C5 Pentacle Structures: A Localization‐Delocalization Matrices Approach
Source: ChemistryOpen. 2024 May 16;13(9):e202300277. doi: 10.1002/open.202300277 (PMC11633349; doi:10.1002/open.202300277)
Supplement: Supplementary file 1 — Supporting Information [file OPEN-13-e202300277-s001.pdf]

ChemistryOpen

Supporting Information

C_5 Pentacle Structures: A Localization-Delocalization Matrices Approach

Julien Pilmé* and Riccardo Spezia*

LDM MATRICES

$$\text{Linear} = \begin{pmatrix} 5.249 & 0.601 & 0.601 & 0.040 & 0.040 \\ 0.601 & 3.717 & 0.161 & 0.970 & 0.117 \\ 0.601 & 0.161 & 3.717 & 0.117 & 0.970 \\ 0.040 & 0.970 & 0.117 & 4.972 & 0.068 \\ 0.040 & 0.117 & 0.970 & 0.068 & 4.972 \end{pmatrix}$$

$$\text{W}_1 = \begin{pmatrix} 4 & 1 & 1 & 0 & 0 \\ 1 & 4 & 0 & 1 & 0 \\ 1 & 0 & 4 & 0 & 1 \\ 0 & 1 & 0 & 4 & 0 \\ 0 & 0 & 1 & 0 & 4 \end{pmatrix}$$

$$\text{W}_2 = \begin{pmatrix} 3 & 0.5 & 0.5 & 0 & 0 \\ 0.5 & 5.5 & 0 & 1 & 0 \\ 0.5 & 0 & 5.5 & 0 & 1 \\ 0 & 0.5 & 0 & 5.5 & 0 \\ 0 & 0 & 1 & 0 & 5 \end{pmatrix}$$

$$\text{W}_3 = \begin{pmatrix} 7 & 0.5 & 0.5 & 0 & 0 \\ 0.5 & 5 & 0 & 0.5 & 0 \\ 0.5 & 0 & 5 & 0 & 0.5 \\ 0 & 0.5 & 0 & 4.5 & 0 \\ 0 & 0 & 0.5 & 0 & 4.5 \end{pmatrix}$$

$$\text{Pentacle-Irregular} = \begin{pmatrix} 4.070 & 0.355 & 0.355 & 0.566 & 0.566 \\ 0.355 & 4.308 & 0.069 & 0.758 & 0.086 \\ 0.355 & 0.069 & 4.308 & 0.086 & 0.758 \\ 0.566 & 0.758 & 0.086 & 4.470 & 0.585 \\ 0.566 & 0.086 & 0.758 & 0.585 & 4.470 \end{pmatrix}$$

$$\text{Pentagon-Regular} = \begin{pmatrix} 4.008 & 0.853 & 0.853 & 0.142 & 0.142 \\ 0.853 & 4.008 & 0.142 & 0.853 & 0.142 \\ 0.853 & 0.142 & 4.008 & 0.142 & 0.853 \\ 0.142 & 0.853 & 0.142 & 4.008 & 0.853 \\ 0.142 & 0.142 & 0.853 & 0.853 & 4.008 \end{pmatrix}$$

$$\text{Cyclopentadienyl (C}_5\text{ cycle)} = \begin{pmatrix} 4.270 & 0.662 & 0.619 & 0.012 & 0.039 \\ 0.662 & 4.170 & 0.144 & 0.072 & 0.564 \\ 0.619 & 0.144 & 4.379 & 0.444 & 0.029 \\ 0.012 & 0.072 & 0.444 & 4.527 & 0.542 \\ 0.039 & 0.564 & 0.029 & 0.542 & 4.470 \end{pmatrix}$$

$$\text{Bipyramid Trigonal} = \begin{pmatrix} 4.774 & 0.378 & 0.378 & 0.269 & 0.378 \\ 0.378 & 4.354 & 0.378 & 0.386 & 0.386 \\ 0.378 & 0.386 & 4.354 & 0.378 & 0.386 \\ 0.269 & 0.378 & 0.378 & 4.774 & 0.378 \\ 0.378 & 0.386 & 0.386 & 0.378 & 4.354 \end{pmatrix}$$

$$\text{Tetrahedron} = \begin{pmatrix} 5.188 & 0.431 & 0.431 & 0.431 & 0.431 \\ 0.431 & 4.574 & 0.257 & 0.257 & 0.257 \\ 0.431 & 0.257 & 4.574 & 0.257 & 0.257 \\ 0.431 & 0.257 & 0.257 & 4.574 & 0.257 \\ 0.431 & 0.257 & 0.257 & 0.257 & 4.574 \end{pmatrix}$$

Table S1: Structural and energy parameters of C_5 structures optimized at the PBE0/aug-cc-pVTZ level of theory. The carbons numbering is given in Figure 1. (a) Three imaginaries frequencies have been found.

**Molecule C_5**	Symmetry	ΔE(kcal/mol)	Type	R_{12} (Å)	R_{23} (Å)	R_{14} (Å).
Linear	$D_{\infty h}$	0.0	minimum	1.278	2.557	2.561
Pentacle-Irregular	planar, C_{2v}	51.4	minimum	1.607	3.171	1.450
Bipyramid trigonal	D_{3h}	81.7	minimum	1.489	2.015	1.859
Pentagon-Regular	planar, D_{5h}	192.5	saddle point$^{(a)}$	1.382	2.237	2.237
Tetrahedron	T_d	254.2	saddle point$^{(a)}$	1.418	2.315	2.315
$C_5H_5^-$	planar, D_{5h}		minimum			

Table S2: Internal (C1-C2) and external (C2-C4) distances for linear C_5 as obtained in the present work and in literature. The difference between external and internal C-C distances is also reported, as well as the rotational constant.

Method	C1-C2	C2-C4	Δ	B (GHz)	Reference
M062X/aug-cc-pVTZ	1.2787	1.2797	+0.0010	2.574066	This work
PBE0/aug-cc-pVTZ	1.2787	1.2829	+0.0042	2.568938	This work
CCSD(T)/cc-PVDZ	1.3057	1.3164	+0.0107		Ref.[S1]
MP2/D95*	1.280	1.277	-0.003		Ref.[S2]
MP2/D95	1.282	1.287	+0.005		Ref.[S2]
B3LYP/6-31G(d)	1.292	1.287	+0.005		Ref.[S3]
B3LYP/aug-cc-pVTZ				2.5704	Ref.[S4]
CASPT2/ANO-L	1.279	1.291	+0.012	2.55	Ref.[S4]
CCSD/cGTOs	1.27788	1.27523	-0.00265	2.5833	Ref.[S5]
CCSD(T)/cGTOs	1.28190	1.28959	+0.007	2.5506	Ref.[S5]
Exp.				2.5487	Ref.[S6]

Table S3: Vibrational harmonic frequencies (in cm^{-1}) obtained for the linear structure with M06-2X/au-cc-pVTZ and PBE0/aug-cc-pVTZ (unscaled frequencies). In parenthesis we report the IR intensities (in kM/mol).

Symmetry	M06-2X	PBE0	B3LYP[S4] aug-cc-pVTZ	CCSD(T)[S1] cc-pVDZ	Exp.
π_u	102 (12)	115 (14)	119		101$^{(a)}$; 118$^{(b)}$
π_g	193 (0)	215 (0)	224		222$^{(a)}$; 218$^{(b)}$
π_u	573 (6)	567 (4)	563		512$^{(a)}$
σ_g	807 (0)	802 (0)	797	770	798$^{(a)}$
σ_u	1518 (131)	1503 (137)	1493	1436	
σ_g	2076 (0)	2055 (0)	2039	1967	
σ_u	2267 (4087)	2271 (3066)	2249	2212	2169$^{(a)}$

$^{(a)}$ From Ref.;[S7] $^{(b)}$ From Ref.[S8,S9]

Table S4: Vibrational harmonic frequencies (in cm^{-1}) obtained for the cyclic Pentacle-Irregular structure with M06-2X/au-cc-pVTZ and PBE0/aug-cc-pVTZ (unscaled frequencies). The three rotational constants (A, B, C, in GHz) are also shown. In parenthesis we report the IR intensities (in kM/mol).

	M06-2X	PBE0
ν_1	141 (8)	98 (11)
ν_2	331 (6)	317 (6)
ν_3	376 (0)	372 (0)
ν_4	510 (0)	499 (0)
ν_5	853 (71)	805 (61)
ν_6	898 (11)	871 (12)
ν_7	1357 (0)	1326 (1)
ν_8	1528 (59)	1497 (49)
ν_9	1728 (29)	1703 (27)
A	28.69741	28.52812
B	7.03847	7.00567
C	5.65219	5.62447

Table S5: Vibrational harmonic frequencies (in cm^{-1}) obtained for the Bipyramid trigonal structure with M06-2X/au-cc-pVTZ and PBE0/aug-cc-pVTZ (unscaled frequencies). The three rotational constants (A, B, in GHz) are also shown. In parenthesis we report the IR intensities (in kM/mol).

Symmetry	M06-2X	PBE0	B3LYP/aug-cc-pVTZ[S4]
E”	495(0)	543 (0)	425
E’	580 (17)	583 (17)	583
A_1’	684 (0)	701 (0)	706
A_2”	850 (96)	859 (88)	808
E’	1080 (5)	1053 (1)	1007
A_1’	1267.7377 (0)	1269 (0)	1236
A	11.232	11.205	11.12
B	10.403	10.378	10.234

References

(S1) Martin, J.; Taylor, P. Structure and Vibrations of Small Carbon Clusters from Clupled-Cluster Calculations. *J. Phys. Chem.* **1996**, *100*, 6047–6056.

(S2) Ewing, D.; Pfeiffer, G. Structures of C5. *Chem. Phys. Lett.* **1987**, *134*, 413–417.

(S3) Dua, S.; Bowie, J. Atom Scrambling of Linear C5 in the Gas Phase: a Joint Experimental and Theoretical Study. *J. Phys. Chem.* **2002**, *106*, 1374–1380.

(S4) Massó, H.; Veryazov, V.; Malmqvist, P.-; Roos, B. O.; Senent, M. L. Ab initio characterization of C5. *J. Chem. Phys.* **2007**, *127*, 154318.

(S5) Botschina, P. The equilibrium geometry and some spectroscopic constants of C5 from large-scale ab initio calculations. *J. Chem. Phys.* **1987**, *101*, 853.

(S6) Moazzen-Ahmadi, N.; Flatt, S.; McKeller, A. . *Chem. Phys. Lett.* **1991**, *186*, 291.

(S7) Arnold, D. W.; Bradforth, S. E.; Kitsopoulos, T. N.; Neumark, D. M. Vibrationally resolved spectra of C2–C11 by anion photoelectron spectroscopy. *J. Chem. Phys.* **1991**, *95*, 8753–8764.

(S8) Moazzen-Ahmadi, N.; McKellar, A. R. W.; Amano, T. Diode laser spectroscopy of gas phase C5: The 3 fundamental and associated hot bands. *J. Chem. Phys.* **1989**, *91*, 2140–2147.

(S9) Moazzen-Ahmadi, N.; McKellar, A.; Amano, T. Laboratory observation of the rotation-vibration spectrum of gas-phase C5. *Chem. Phys. Lett.* **1989**, *157*, 1–4.
